# Global research trends on cardiac troponin and physical activity among pediatric populations: a bibliometric analysis and science mapping study

**DOI:** 10.3389/fped.2024.1285794

**Published:** 2024-02-05

**Authors:** Vicenç Hernández-González, Enric Conesa-Milian, Carme Jové-Deltell, Álvaro Pano-Rodríguez, Alejandro Legaz-Arrese, Joaquin Reverter-Masia

**Affiliations:** ^1^ Human Movement Research Group (RGHM), University of Lleida, Lleida, Spain; ^2^ Physical Education and Sport Section, University of Lleida, Lleida, Spain; ^3^ Section of Physical Education and Sports, Faculty of Health and Sport Sciences, University of Zaragoza, Zaragoza, Spain

**Keywords:** cardiac troponin, pediatric, bibliometrics, productivity, network analysis, Web of Science, sports science

## Abstract

**Background:**

Cardiac troponin (cTn) is a reliable marker for evaluating myocardial damage. cTn is a very specific protein involved in myocardial injury, and it is a key factor in the diagnosis of coronary syndromes. Bibliometric analysis was applied in the present work, with the main goal of evaluating global research on the topic of cardiac troponin in pediatric populations.

**Methods:**

Publications about cardiac troponin and physical activity in pediatric populations were retrieved from the Social Sciences Citation Index (SSCI) and the Science Citation Index Expanded (SCIE) of the Web of Science Core Collection, and they were then analyzed. The study was able to identify the key bibliometric indicators, such as publications, keywords, authors, countries, institutions, and journals. For the analysis, VOSviewer, R-based Bibliometrix (4.2.2), and MapChart were used.

**Results:**

Initially, 98 documents were identified; however, once inclusion and exclusion criteria were applied, the number of documents decreased to 88. The search yielded 79 original research articles and 9 reviews, almost all of which were published in the past 2 decades. The total number of citations (Nc) of the retrieved publications was 1,468, and the average number of citations per article (Na) was 16.68. In general, 508 authors were found to have participated in research about troponin; they were associated with 256 institutions, and their work was published in 65 different journals from around the world. The authors hailed from 30 countries and/or regions. The year 2022 was the most productive year for the publication of the selected documents. The bibliometric analysis provided information regarding levels of cooperation among authors and institutions. In fact, China, the United States, and England were the most productive nations, and the journal with the greatest number of publications on the topic was Pediatric Cardiology.

**Summary:**

The number of publications and the trend line show that research on this topic has not yet reached a stage of maturity. There are referent investigators, countries, and institutions that have laid the foundations for subsequent studies on the analyzed topic.

## 1 Introduction

We have known for some time that continuous exercise produces changes and benefits in the heart (1) and that increasing cardiac capacity is positive. However, it has also been proven that, after engaging in exercise, athletes (2), including soccer players (3), rowers (4), swimmers (5), triathletes (6), and basketball players (7), experience higher levels of troponin, a protein that signals cardiac damage.

Troponin (Tn) is a regulatory protein responsible for the contraction of striated cardiac and skeletal muscles (8). This protein can be found in thin filaments, and it is made up of three subunits: C, T, and I. Troponin C (TnC) is responsible for binding to Ca2+, troponin T (TnT) attaches to tropomyosin, and troponin I (TnI) decreases the affinity of TnC to Ca2+, thereby inhibiting the contraction of myofilaments (9). Cardiac troponin (cTn) is a specific biomarker for heart damage and is the leading criterion for diagnosing myocardial injury (10, 11). The cTn isoforms of cardiac troponins T and I (cTnT and cTnI) are highly specific to myocardial cell damage and are key factors in the diagnosis of acute coronary syndromes and necrosis (12). In recent years, highly sensitive assays have been developed to determine the blood concentrations of cTnI and cTnT (hs-cTnI and hs-cTnT).

More advanced and sensitive tests have replaced the standard ones. These new tests, referred to as “high-sensitivity”, can detect extremely low levels of cTn in 99% of the population, with a small amount of variation, >10% (13–15). They can also identify cTn levels in at least 50% of healthy individuals when they are at rest. However, this higher sensitivity can sometimes lead to false-positive results, meaning that the tests may indicate a problem, such as an acute myocardial infarction (AMI), when the elevated level was caused by something else, such as physical exercise (16–18). The fourth definition, or clinical criterion, for myocardial infarction (MI) “denotes the presence of acute myocardial injury detected by abnormal cardiac biomarkers in the setting of evidence of acute myocardial ischaemia” (19). Nevertheless, to diagnose myocardial injury, doctors look for elevated cTn levels above the upper reference limit, which represents the 99th percentile (URL), a value that is used as a reference range; this situation is considered acute if there is an increase and/or decrease in cTn values (19).

Numerous investigations have shown that, in healthy individuals, the blood concentration of cTn increases after prolonged exercise, (e.g., running a marathon) (20, 21) and after shorter exercise sessions (30–60 min) (2, 4). In addition to the duration of the exercise, it should be noted that the intensity of the exercise sessions is also very important in assessing the increase in troponin. In fact, in the study by Peretti et al. (22) it has been observed that short, high-intensity exercise caused an elevation in cardiac biomarkers in 62% of the population. It has also been observed that the degree of training can influence troponin release. The percentage of subjects with postexercise cTn levels above the URL ranged from 0% (23) to 100% (24) in individual studies. Controlled studies that use high-sensitivity assays and perform multiple blood draws after exercise report that a release of cTn is evidenced in practically all subjects. They also observed high individual variability in peak values during recovery (25, 2). The clinical relevance of the postexercise cTn increase in healthy subjects is not entirely clear. In this topic, it is important to highlight that the magnitude and kinetics of cTn after exercise are different from thoseobserved in acute myocardial infarction (19, 26). Thus, the data from different studies reflects consistent kinetics in all subjects with a rapid increase in cTn during the first hours after exercise, with most subjects reaching the maximum value after 3–6 h and with values returning close to baseline levels at 24 h postexercise (25, 2, 4, 6). Furthermore, in these studies, the increase in cTn occurred in the absence of clinical signs and symptoms. It is also important to note that the release of cTn is evidenced after short- and long-duration efforts, which are known to induce beneficial effects (27).

Cardiac troponin appears to be elevated in children and adolescents after they have engaged in aerobic exercise, which is a finding that is consistent across most studies (28, 29). In this context, it is relevant to review all the information about any investigation in which the main goal was the study of cardiac biomarker liberation (29).

To review an extensive source of information on any research topic, numerous studies have indicated bibliometric studies as effective for this type of analysis (30). Bibliometrics are used in the field of library and information science. As a method of statistical analysis, they play an important role in impact and tendency analyses (30). Additionally, they allow for the analysis of a significant amount of scientific literature within a given research area (31). Moreover, using bibliometrics can be a complementary orientation strategy for research, and it can play a significant function in decision making in the development of the research (32).

Furthermore, bibliometrics involve the analysis of the cooperation, citations, journals, and institutions that make research visible (33). These types of indicators allow for the identification and definition of the characteristics of the most productive authors and the existing collaborations between them; the centers that generate the research; the intrainstitutional and interinstitutional collaborations; the primary sources in which the works are published; their productivity, concentration, or dispersion; their national and international diffusion; and the repercussions and impact they have on subsequent work (34, 35).

Understanding the current status, areas of focus, productivity growth, and scientific collaboration on heart damage and physical exercise will allow established researchers to expand their circles of contacts, and encourage broader participation in discussions and forums for the exchange of ideas and the expansion of work groups and networks, or the integration of new members into them (36–38).

In recent years, bibliometric mapping has been widely analysed within the field of public health (39–43). Furthermore, there is a recently published, retrospective study on heart failure biomarkers (44) that presents a bibliometric analysis that aims to assess the state of research on biomarkers and heart failure. However, to our knowledge, no bibliometric studies have been carried out on cardiac troponin as specifically linked to the field of sports science in pediatric populations. Understanding the current status, areas of focus, and future perspectives on cardiac troponin will help us to explore the intrinsic relationship between the discipline’s knowledge structure and its development mechanisms in the field of health, providing deeper insight into the role that cardiac troponin plays. Doing so will also assist in the discovery of innovative strategies for clinical heart disease prevention and treatment problems.

The main objective of the present study was to analyse systematically the scientific findings on cardiac troponin in pediatric populations, providing a bibliometric view of research in the field of cardiac troponins. This bibliometric analysis will help identify relevant and potentially useful publications of cardiac biomarker measurements for cardiovascular risk assessment in the pediatric population. The bibliometric study will recognize the most influential authors, journals, institutes, articles and countries in the development of research on cardiac biomarkers. At the end of the document, relevant aspects of the research are discussed, as well as findings that guide future research.

## 2 Materials and methods

### 2.1 Search strategy and eligible criteria

Our raw data were retrieved and downloaded from the Web of Science Core Collection (WoSCC) Science Citation Index Expanded (SCIE) and Social Sciences Citation Index (SSCI) developed by Thomson Scientific. The Web of Science consists of many high-quality, high-impact scientific studies, making it the most comprehensive and inclusive collection of information available worldwide (33, 45). Due to differences in the citation data in each database, no database used for bibliometric studies is considered to be better (45, 46). The search strategy was performed using MeSH terms, which were employed in the following manner: TS = (Troponin OR Troponin Complex OR TnT OR hs-cTn OR cTn) AND TS = (Children OR Adolescence* OR Teen* OR Youth* OR Female Adolescent OR Male Adolescent) AND TS = (Exercise OR Physical Activity OR Physical Exercise OR Acute Exercise OR Isometric Exercise OR Aerobic Exercise OR Exercise Training OR Sport). In this manner, we retrieved all of the articles and reviews on troponin in children and adolescents as related to physical exercise that were published online between 1900 and 2023 from the WoSCC SCIE and SSCI.

To reduce the risk of bias, two independent authors obtained the information from all the documents on the same day (24th of February 2023).

**Figure 1:**
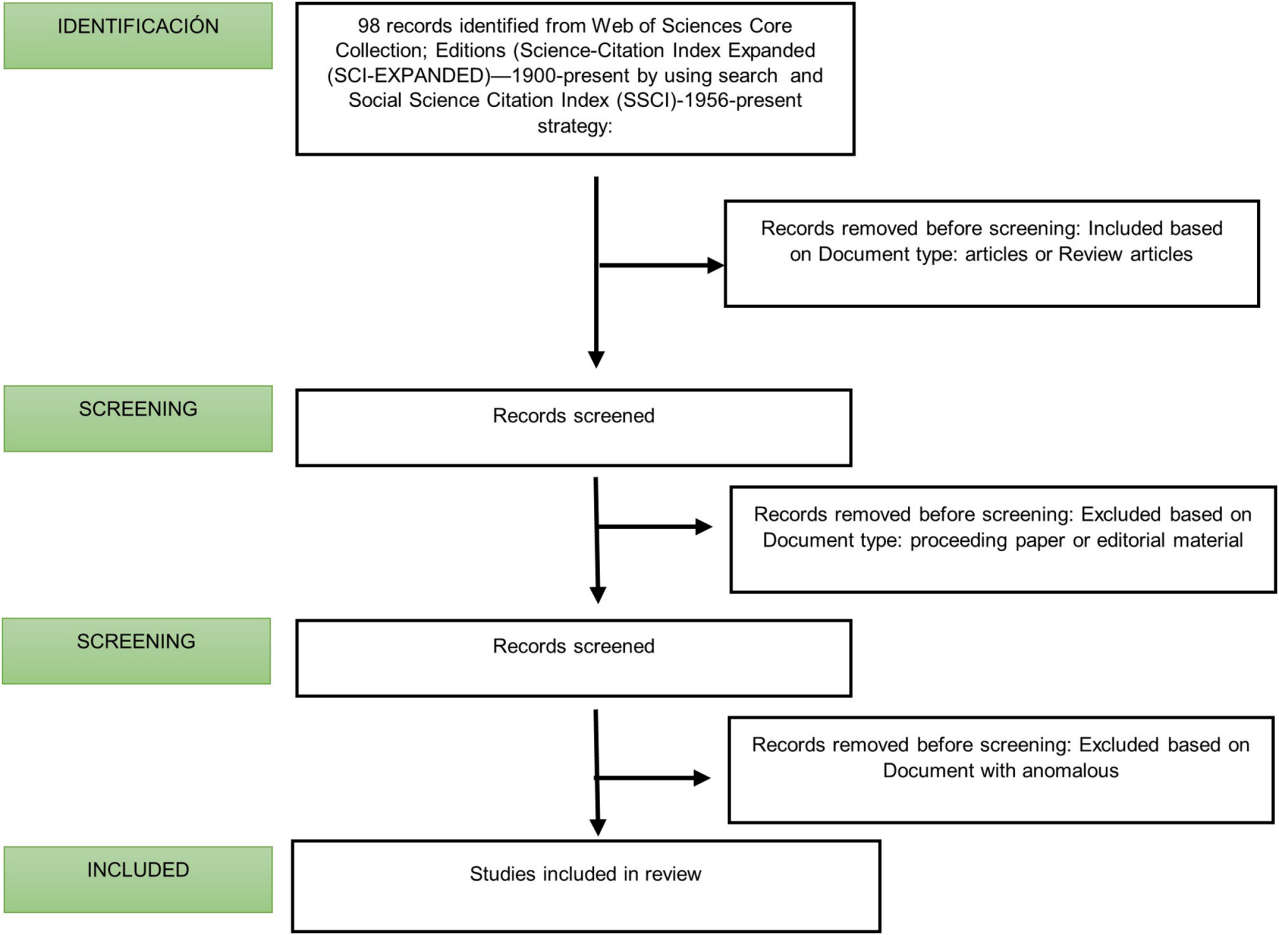
Flowchart of the present study.

Using Web of Science, the documents that met the inclusion and exclusion criteria were selected and exported. The search strategy process and study selection are detailed in Figure 1. For the analysis, only articles or reviews were taken into consideration, which led to the exclusion of one document based on the document type; we also excluded proceeding papers and editorial material, which led to the exclusion of seven documents. Those documents that, after review by the two authors, did not comply with the MeSH terms used in the search strategy were also excluded. Finally, 88 original articles and reviews were included in the analysis and further analyzed and visualized. Later, they were exported to an Excel document where the following information was recorded: the number of citations; name of the journal; year of publication; first and last names of the author and co-authors; total number of authors; geographical location, origin, and associated institute of the authors; title of the article; type of document (article or review); abstract; and the corresponding author. For the analysis of the authors, all individuals who participated in the study were counted. In the bibliometric analysis by country, the country of each author who participated in the study was taken into account, and the citations received were counted. Citations received by a country more than once were not counted if several authors from different institutions (but from the same country) had participated in the same study. The number of articles per country was counted as long as there was an author from the country in the study.

### 2.2 Bibliometric analysis

In this study, objective and evaluative bibliometrics were used to visualize and analyze findings on cardiac troponin research. Objective bibliometrics measure the quantity of literature and the number of citations, and they also involve citation analyses (47). The productivity, impact, and quality of a publication are expressed as the number of publications (Np), the number of citations (Nc), and the mean number of citations (Na). Evaluative bibliometrics provide quantitative evaluations of countries’, authors’, journals’, and institutions’ field contributions, and their measurable index is known as their h-index (48). This type of analysis can identify the articles that influence the history of a field, and it can uncover current research hotspots and future trends (49).

### 2.3 Statical analysis

We used SPSS 27.0 software (IBM, USA) to perform the correlation analysis. Microsoft Office Excel was used to conduct a linear regression analysis to evaluate the publication trend over time. A polynomial model was applied to predict the increase in publications. We used a popular bibliometric analysis tool, VOSviewer 1.6.18 (CWTS, The Netherlands) (50) for cooperative network identification and keyword co-occurrence analysis. We also used the Bibliometrix tool in R (4.2.2). The R package is intended to be used in quantitative scientometrics and informetrics (51). The tool offers various means for importing bibliographic data from Clarivate's Web of Science. Furthermore, the bibliometric packages permit the classification and analysis of vast quantities of historical research data from a defined period so as to acquire metadata from the database.

The analysis identified information based on the concurrent relation and map distance. The interpretation of the maps was simple due to the colors, sizes, and distance classifications (clusters) of the evaluated terms. In addition, the software can generate visual maps of knowledge. We also used the MapChart program to generate a personalized map of different regions of the world, accompanied by colors and descriptions.

## 3 Results

The study flowchart, shown in Figure 1, includes studies that were published between 1900 and 2023, records that were identified from the Web of Science Core Collection, and specifically from within the Social Sciences Citation Index (SSCI) and the Science Citation Index Expanded (SCIE) by using the search strategy. After applying the strategy, the search topic produced 98 documents.

### 3.1 The global publishing landscape

We analyzed a total number of 88 documents, divided in 79 articles and 9 reviews. Virtually all of the works were published within the last two decades. The total number of citations (Nc) of all of the retrieved publications was 1,468, and the average number of citations per article (Na) was 16.68. A total of 508 authors from 30 countries had published one or more papers on troponin. The 88 papers were published in 65 journals.

**Figure 2:**
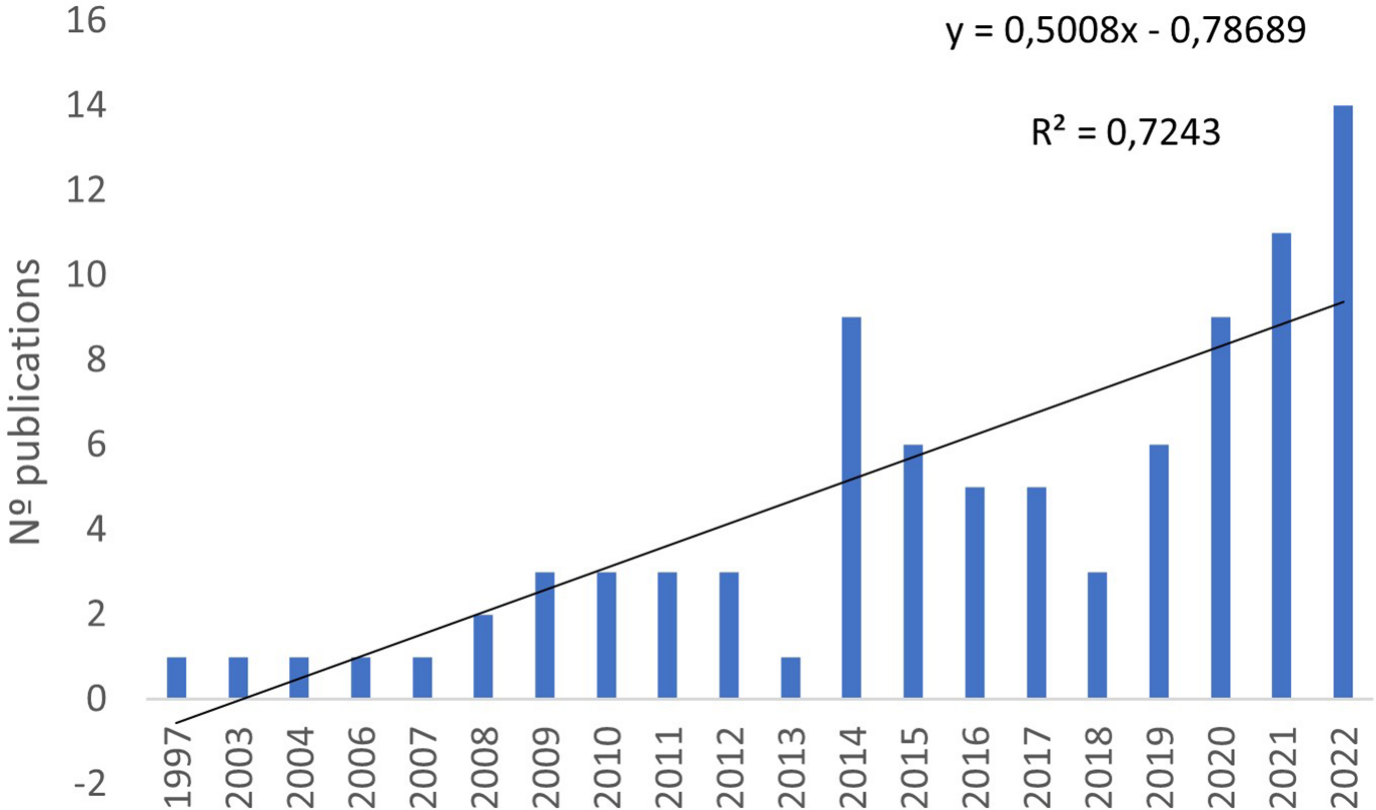
Distribution pattern of the articles (number of articles per year).

The works were published between 1997 and 2022, of which 85% were published after 2010. We performed an analysis of publication trends using 5-year intervals based on a ranking of the publication dates. Between 2018 and 2022, 43 documents were published, almost 50% of the total number of publications. The years 2021 (*n* = 11) and 2022 (*n* = 14) were the years of greatest production. There can be seen a visible improvement in the quantity of the data, and exponential growth can be observed in recent years. According to our linear regression analysis, there exists a positive correlation between the number of publications per year and the year of publication (*R*^2^ = 0.724, *p* < 0.001) (Figure 2).

**Figure 3:**
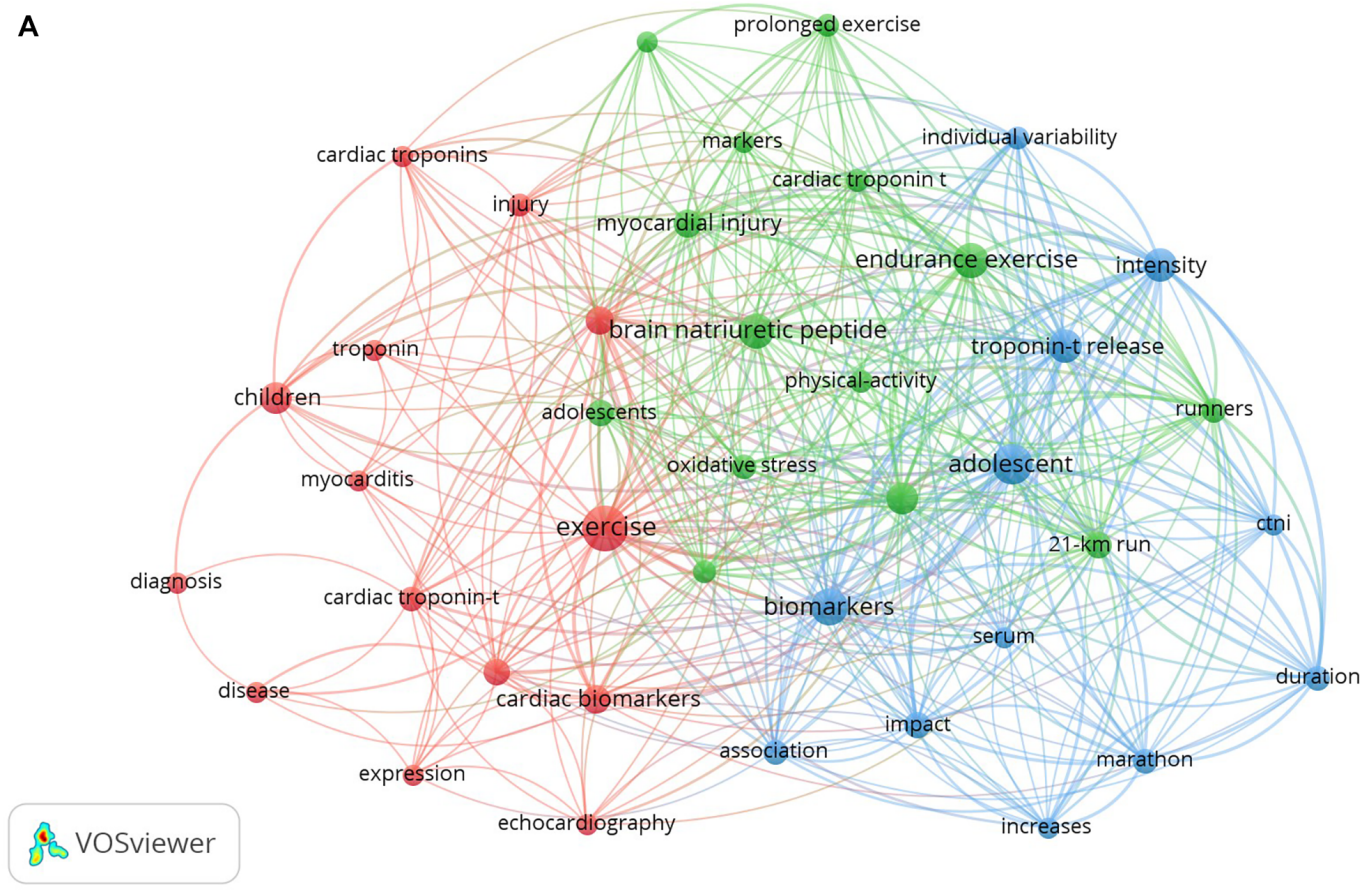
(**A**) The co-occurrence network of keywords. The size of the nodes indicates the frequency of occurrence. The curves between the nodes represent their co-occurrence in the same publication. The smaller the distance between two nodes, the higher the number of co-occurrences of the two keywords; (**B**) the relationship between the terms in time is illustrated. The color of the terms indicates their average publication period. This value is calculated by taking the average of the years of publication of all publications; (**C**) word Cloud for Cardiac Troponin topic.

The analysis of publication terms can provide an idea about the principal research topics and trends. This was developed using VOSviewer software, taking into account the title, keywords, and abstract. Terms that appeared in at least five publications were considered, and 40 terms were selected for inclusion in the net. The results from this analysis are presented in Figure 3, and there are three distinct clusters. The red group, or first node, is related to terms such as “exercise”, “children”, “cardiac troponin”, and “cardiac biomarkers”, among others, with “exercise” being the most related term. The green group, a part of the second node, is related to terms such as “brain natriuretic peptide”, “endurance exercise”, “NT-proBNP”, and “runners”. In this second node, the term “brain natriuretic peptide” was the one that presented a stronger relationship. The third node, shown in blue, represents all of the terms related to “adolescent”, “biomarkers”, “troponin-t release” and “intensity”, with the term “adolescent” being the most related. The most frequent words were “exercise”, “adolescent”, “biomarkers”, “brain natriuretic peptide”, and “endurance exercise” (see Figure 3A).

**Figure:**
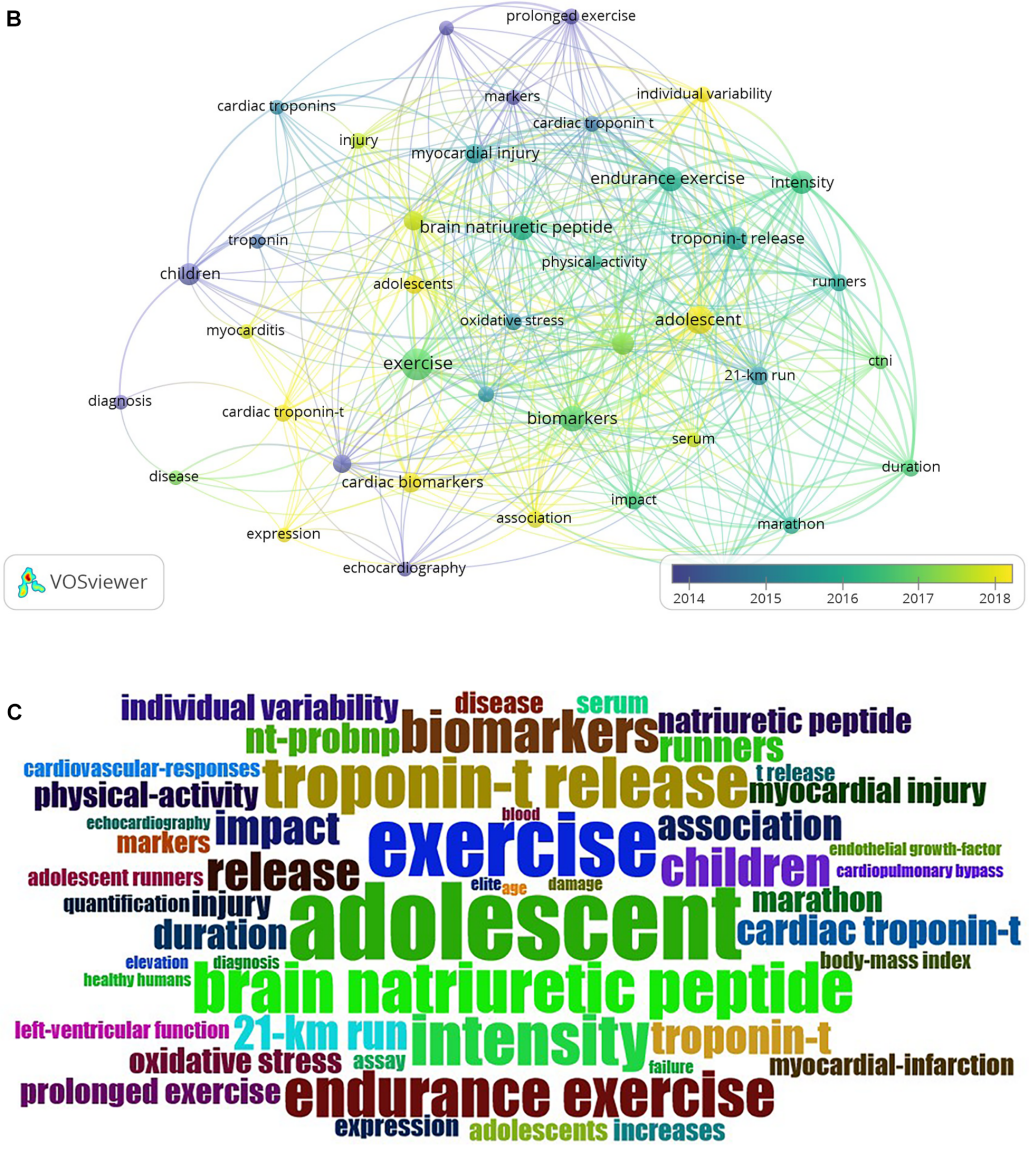


The most used terms were identified in the range from 2014 to 2018. The oldest terms are presented in blue, while the most current terms are presented in yellow. Based on the range of colors and the size of the circle, the most frequently recurring terms appeared between 2016 and 2017. The clusters with current terms were those related to “adolescent”, “cardiac troponin-t”, and “cardiac biomarkers”. The cluster related to the terms “children”, “troponin”, “diagnosis”, and “cardiac troponin” refers to older terms. Some terms, such as “troponin” and “cardiac troponin” have evolved into terms that are more specific; such as “cardiac troponin-t”, “cardiac biomarkers”, and “NT-proBNP”. In the same way, it seems that the most recent research has focused on the study of maturational stages, such as adolescence, as compared to younger ages, as was the case in 2014 or 2015 (Figure 3B).

Figure 3C shows a word cloud of the analyzed subject. As expected, the cloud illustrates the strong interaction between the words “adolescent”, “exercise”, “troponin-t release”, and “brain natriuretic peptide”.

### 3.2 Authors and bibliometric analysis of the co-authorship

A total of 508 authors contributed articles on the troponin's topic. The number of authors on each article ranged from 1 to 22 (mean = 7.43). Our analysis of the 10 most productive authors, based on their number of articles, but regardless of their authorship positions, showed that J.L. Nie, K. George, and J. Reverter-Masia were the authors with the greatest number of articles on the topic.

J.L. Nie, from the People's Republic of China, had 442 citations—the maximum number—with 18 articles listed, and an h-index of 10 in relation to the topic of troponin. The average number of citations per article was 23. However, K. George, from England, published 17 papers, with a total of 342 citations, resulting in an average of 20 citations per article and an h-index of 11. The third most prolific researcher was J. Reverter-Masia. Hailing from Spain, this researcher published 10 documents with more than 110 total citations, resulting in an h-index of 6 (see Table 1). We found that the total number of citations was related negative to the number of authors (rs = −0.051, *p* < 0.001).

**Table 1:** The top 10 authors with the most documents on the topic of troponin.

Author	Np	h-index	First author	Last author	Co-author	Nc
Nie, J.L.	18	10	5	1	12	422
George, K.	17	11	0	5	12	342
Reverter-Masia, J.	10	6	0	7	3	110
Legaz-Arrese, A.	9	6	3	1	5	108
Tong, T.K.	9	8	1	1	7	288
Shi, Q.D.	9	7	0	5	4	225
Lopez-Laval, I.	7	5	1	0	6	84
Zhang, H.F.	7	4	1	0	6	44
Cirer-Sastre, R.	6	4	6	0	0	28
Tian, Y.	6	5	2	1	3	177
Fu, F.	6	5	2	1	3	165

Np, number of publications; Nc, number of citations.

**Figure 4:**
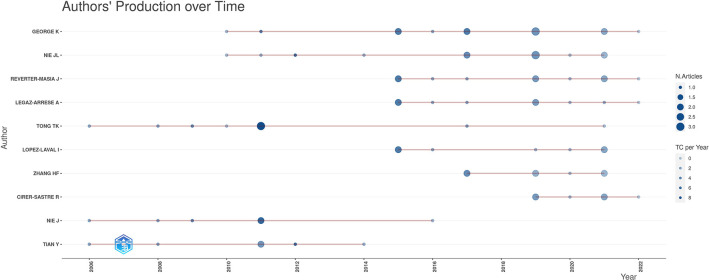
Authors’ level of production over time on the subject of cardiac troponin. The circle sizes in the figure signify the numbers of documents, and the shades of the colors signify the numbers of citations.

The graphical representation of the authors’ level of production on the cardiac troponin's topic over time is displayed in Figure 4. The sizes of the circles in the figure represent the numbers of published articles, and the colors represent the numbers of citations.

**Figure 5:**
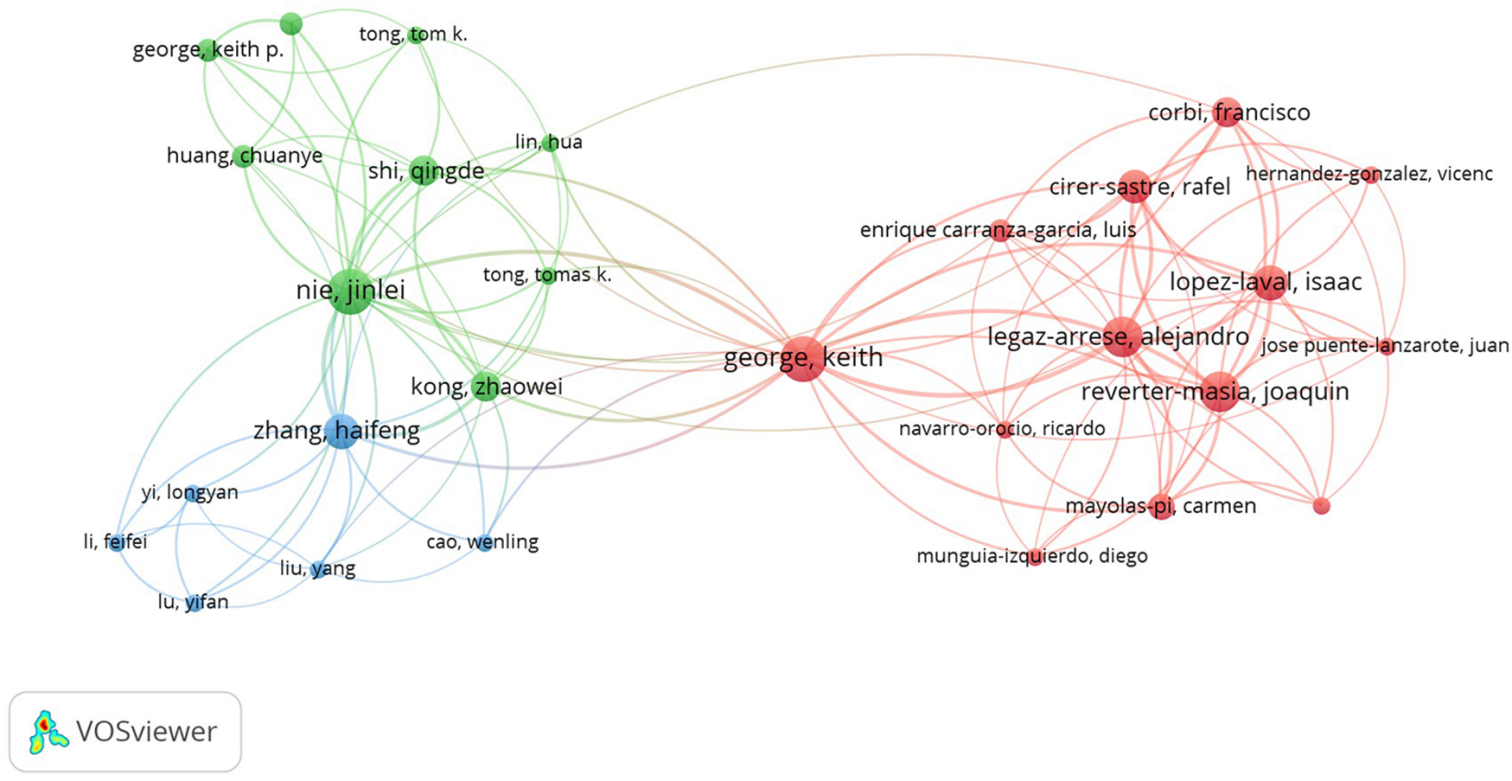
The authors’ collaborative networks. The collaboration map of the authors reflects the scientific research cooperation between them. The circles/nodes signify the authors; the sizes of the circles/nodes signify the numbers of articles. The lines denote the strength of the authors’ collaboration, and each color signifies a cluster.

The pattern of cooperation of each author's publishing was examined using VOSviewer (see Figure 5). There was a high level of collaboration between most of the main authors, creating three cooperative research networks. Authors with a minimum of two papers were considered for analysis. Of the 508 authors, 28 reached the threshold (Figure 4). K. George formed a collaborative network with 21 other researchers. Another cooperative research network was formed by J.L. Nie with 19 researchers. The third cooperative research network was formed around H.F. Zhang with 12 other researchers.

### 3.3 Countries, institutions, and bibliometric analysis of the collaboration

Researchers from a total of 30 countries published 88 articles on the topic analyzed. Table 2 shows the 10 most productive countries, with the People's Republic of China contributing the most, with 24 documents, followed by the USAand England, with 20 articles. These same three countries were also accorded the greatest number of citations. Regarding the average number of citations per article, the Canada was notable among the countries, with an average of 33, 50 citations per article, followed by England, with an average of 20, 80 citations per article.

**Table 2:** The top countries with the greatest number of citations per document on the topic of troponin.

Location	Nc	Np	Na	h-index
People's Republic of China	457	24	19,04	12
England	416	20	20,80	12
USA	406	20	20,30	8
Spain	245	15	16,33	7
Italy	107	8	13,38	5
Australia	58	7	8,29	4
Germany	86	7	12,29	5
France	70	5	14,00	4
Austria	47	4	11,75	3
Canada	134	4	33,50	3

Np, number of publications; Nc, number of citations; Na, average number of citations.

**Figure 6:**
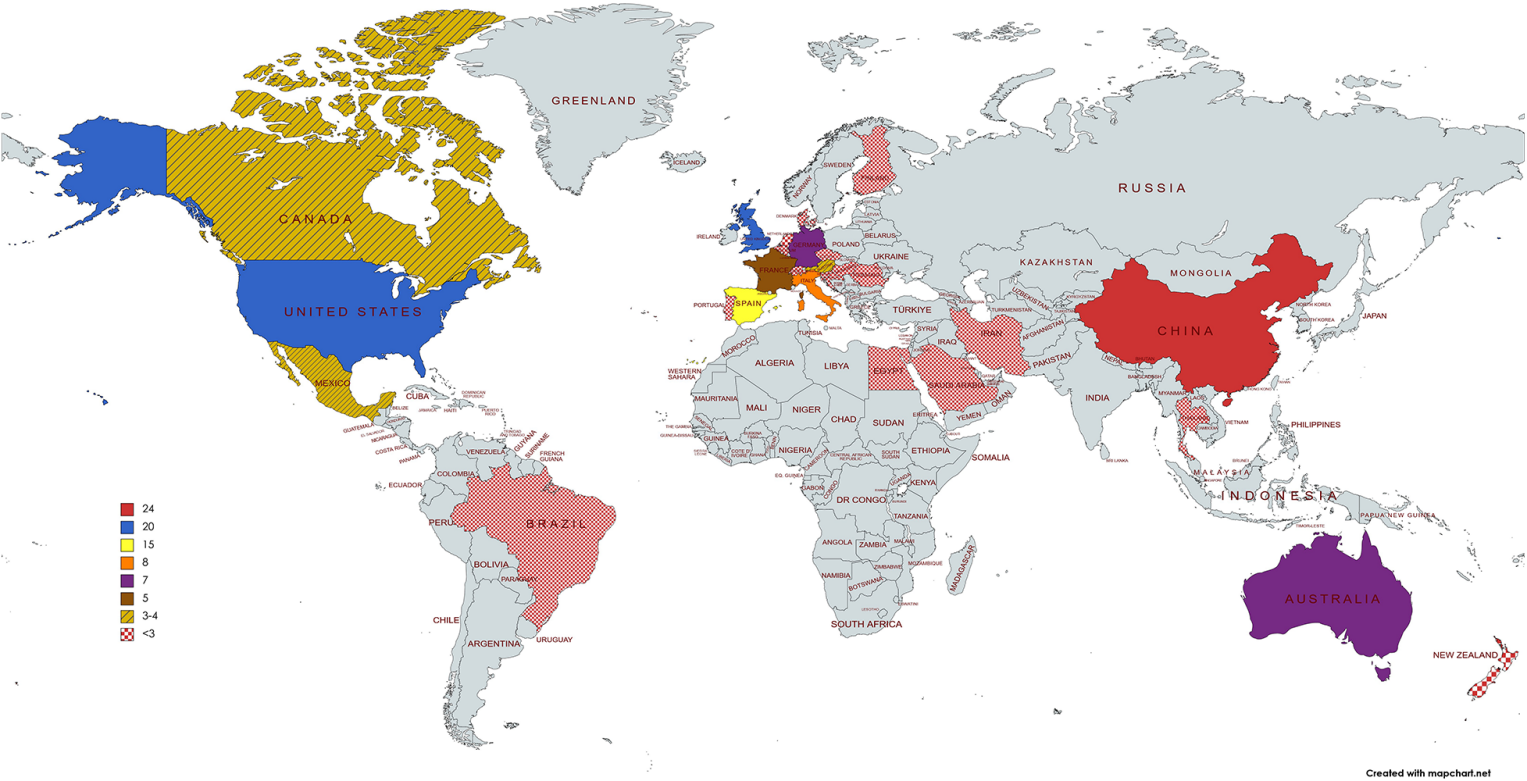
Distribution map showing the numbers of published articles per country (mapChart).

The publications focusing on cardiac troponin were created in 30 different countries, with 19 of those being located in Europe, 4 in Asia, 4 in the Americas, 1 in Africa, and 2 in Oceania. Figure 6 illustrates the global distribution of these countries and regions. The top four countries alone (13.3%) produced almost 60% of the publications. Half of the countries produced only one document. It is important to note that a publication may be related to different countries or institutions due to multiple affiliations or authors as all author affiliations, with respect to both country and institution, were considered. The People's Republic of China produced the greatest quantity of publications, followed by the England and then USA.

**Figure 7:**
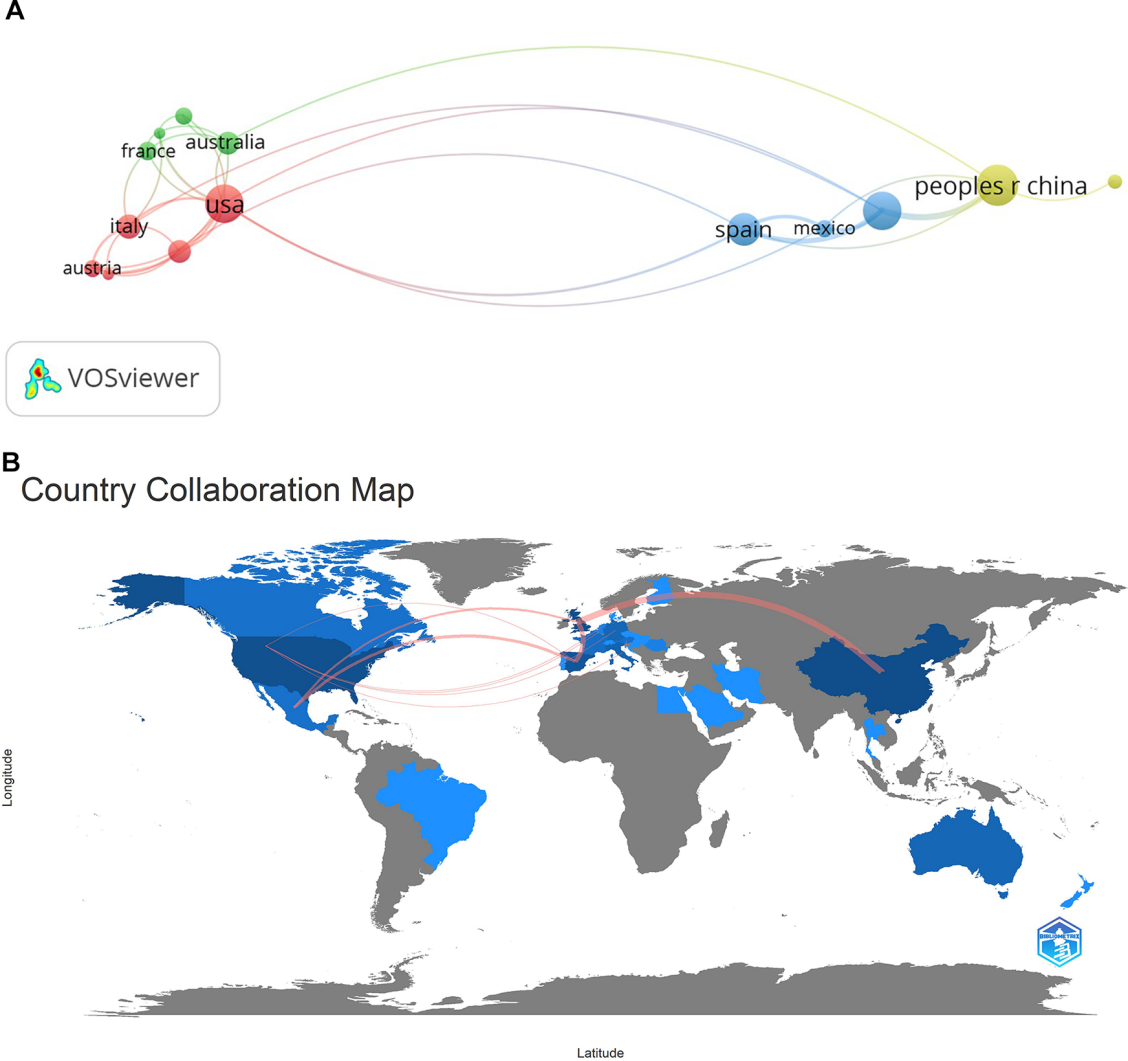
(**A**) Intercountry collaboration networks; (**B**) intercountry collaboration map.

Figure 7A presents our analysis of the collaboration between countries. The analysis was performed using VOSviewer software. Countries with two or more collaborative publications appear in the network. Four collaborative nodes were established. The first node, shown in red, involves five countries, and the USA had the most active associations within this network, with 20 collaborative documents produced. The main research collaborators of the USA included Austria, Germany, Belgium, and Italy. A second, smaller node, shown in green, focuses on Australia, which collaborated on seven papers. Its main research collaborators included Canada, France, and the Netherlands. A third node (shown in blue) centers on England, which produced 20 collaborative documents, with its main connection—to Spain—yielding 15 documents. Finally, a fourth node (shown in yellow) centers around the People's Republic of China with 24 documents; its main research collaborator was Scotland.

The analysis was developed using Bibliometrix software, and it shows the strong level of cooperation between England, Spain, Mexico, and China (see Figure 7B).

In total, 256 institutions participated in the creation of 88 publications according to the available affiliation data. (A publication can be written by several authors, or an author can be affiliated with more than one institution.) The number of institutions per article ranged between 1 and 19. The average number of institutions involved in a collaboration was 3.9 institutions per article. Considering all of the institutions, 80.07% (*n* = 205) participated in only one publication; 14.5% (*n* = 37) participated in two publications; 3.6% (*n* = 9) participated in the creation of three to seven publications. Only five institutions (2%) were involved in the development of 10 or more publications. Liverpool John Moores University and Macao Polytechnic Institute were the two most prolific institutions with 17 publications each. Seven of the ten most productive institutions are located in China, two in Spain, and one in England. The two universities with the highest number of documents listed as the first or last institutions were Lleida University (80% of the documents) and China Institute of Sport Science (66.6%). In addition, the China Institute of Sport Science, together with Liaoning Normal University, were the institutions with the greatest mean for citations by document (35.83 and 33.17 citations per documents, respectively). There was a positivecorrelation between the total number of citations and the number of participating institutions (rs = 0.112, *p* = 0.228).

Most of the institutions are universities, but there is also evidence of the presence of research centers and institutes, and even some private institutions appear to be involved (Table 3).

**Table 3:** The top 10 institutions with the greatest numbers of citations per document.

Institution	Country	Np	No of documents as first or last institution	Nc	Na	h-index
Liverpool John Moores University	England	17	5	342	20.12	11
Macao Polytechnic Institute	China	17	8	384	22.59	9
Hong Kong Baptist University	China	12	4	310	25.83	9
University of Zaragoza	Spain	11	5	212	19.27	7
University of Lleida	Spain	10	8	110	11	6
Hebei Normal University	China	7	2	46	6.57	4
China Institute of Sport Science	China	6	4	215	35.83	6
Liaoning Normal University	China	6	0	199	33.17	6
Beijing Sport University	China	5	3	60	12	4
University of Macau	China	5	1	33	6.6	3

Np, number of publications; Nc, number of citations; Na, average number of citations per publication.

**Figure 8:**
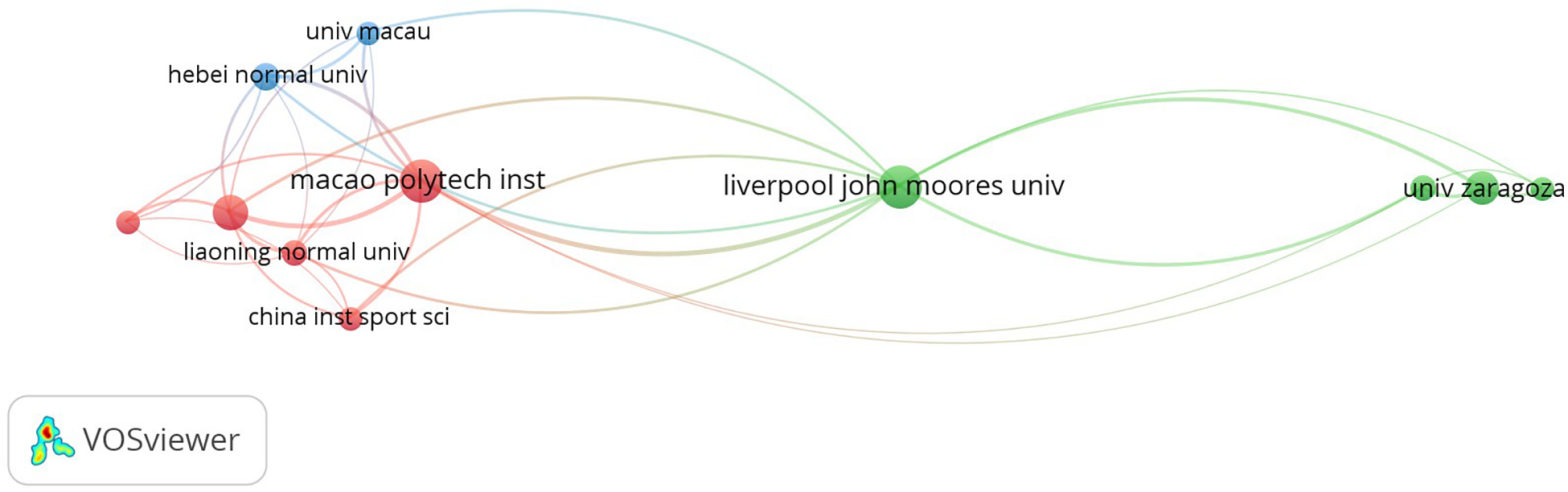
Institutional collaboration networks.

In the collaboration network analysis (see Figure 8), a minimum of three collaborations between institutions were established. A total of 13 institutions reached the threshold, and four cooperation network nodes were formed. In the first of them, the red node, Macao Polytechnic Institute cooperated with institutions including Beijing Sport University, China Institute of Sport Science, Hong Kong Baptist University, and Liaoning Normal University, resulting in collaboration on 17 documents. Liverpool John Moore University Shown in green) had a strong partnership and cooperation with the University of Zaragoza and Lleida University with 17 documents), and the third node (shown in blue), illustrates the strong cooperation between Hebei Normal University and the University of Macau and the Provincial Key Lab of Measurement and Evaluation in Human Movement, resulting in collaboration on seven documents. We observed that Macao Polytechnic Institute and Liverpool John Moores University concentrated their collaboration networks, resulting in a near majority of the scientific production on the subject analyzed.

### 3.4 Journals analysis

Table 4 shows the 10 journals that published the most articles. Pediatric Cardiology was the most productive journal (*n* = 4), followed by Biomarkers, Frontiers in Physiology, International Journal of Environmental Research and Occupational Health, International Journal of Sports Medicine, Pediatric Exercise Science, and Scandinavian Journal of Medicine & Science in Sports, with 3 documents each. A total of 56.8% of the journals published one single document, while the remainder of the journals (*n* = 15) published the remaining 43.2% of the publications.

**Table 4:** The top 10 journals that published articles on the topic of troponin.

Journal title	Np	Nc	Na	Impact factor (2021)	Quartile
Pediatric Cardiology	4	20	5	1.838	Q3
Biomarkers	3	36	12	2.663	Q3
Frontiers in Physiology	3	10	3.33	4.755	Q1
International Journal of Environmental Research and Occupational Health	3	14	4.67	4.614	Q1
International Journal of Sports Medicine	3	105	35	2.997	Q2
Pediatric Exercise Science	3	22	7.33	2.395	Q3
Scandinavian Journal of Medicine and Science in Sports	3	79	26.33	4.645	Q1
Acta Paediatrica	2	20	10	4.056	Q1
Clinical Chemistry and Laboratory Medicine	2	24	12	8.49	Q1
European Journal of Applied Physiology	2	20	10	3.346	Q2

Np, number of publications; Nc, number of citations; Na, average number of citations.

Among the most productive journals, the one with the highest number of citations was International Journal of Sports Medicine (*n* = 105), and the one with the fewest citations was Frontiers in Physiology (*n* = 10).

Of the 65 journals included in the list, 23 of them were ranked in Q1 (35.4%), 23 were ranked in Q2 (35.4%), 10 of them were ranked in Q3 (15.4%), and the remaining 9 were ranked in Q4 (13.8%). Together, 70% of the studies were published in high-impact journals (Q1–Q2).

The IFs of the 65 journals ranged from 0.699 (Heart Surgery Forum) to 41,787 (Intensive Care Medicine).

We found 11 journals with an IF ranging between 0.699 and 1,929, 35 journals that had an IF ranging between 2,141 and 4,997, 12 journals whose impact factor ranged between 5,121 and 8,307, and 7 journals with an IF greater than 10,000.

The top 10 journals published 31.8% of the articles, and they account for more than 68% of the total citations. The journal with the highest number of citations was International Journal of Sports Medicine (*n* = 105), and the mean number of citations per article was 35 citations/article.

## 4 Discussion

In recent years, bibliometric studies have gained great momentum because they provide useful analytical indicators on the evolution of science, new lines of research, or new fields that are developing and are worth investigating. To our understanding, this is the first time that a bibliometric evaluation of the literature related to cardiac troponin in sports science has been carried out. We aimed to obtain a global overview, as well as to understand the foci, themes, and research frontiers in this field.

In this study, we collected data from 80 papers about cardiac troponin in pediatric populations published during the past 2 decades according to the SSCI and SCIE databases. We performed a series of bibliometric analyses and data visualizations using VOSviewer and the Bibliometrix (4.2.2) R package, revealing a growth trend in the number of publications produced in this field per year.

The publication analysis and trend curve on the topic showed that only one document was published prior to the 21st century. Since 2000, the number of documents has been progressively increasing, with 2022 being the year with the greatest level of production. Some research, such as that conducted by Aronson and Fermer (52), or more recently, one produced by Dong et al. (44), has pointed out the significant increase in interest in biomarker studies, showing a clear upwards trend since the end of the last decade. The growth trend in scientific production on cardiac troponin in young people shows that it has not yet reached a stage of maturity, and it could continue to grow in the coming years. In addition, numerous studies suggest that it is necessary to continue conducting research in this area, to establish standardized protocols and to perform more studies with larger populations (53–55).

The keyword co-occurrence analysis revealed that the words “exercise”, “brain natriuretic peptide” and “adolescent” had the highest frequencies of co-occurrence in the research on the topic analysed, creating three clusters. This allows us to ascertain which topics attract more interest in the research community.

Word clouds are another visualization technique, but their goal is completely different, as they provide the user with an overview of the content of a collection of texts. The size of the word (as the source's size) indicates the frequency with which it appears in the text (56). The goal of using a word cloud in an article is to enable searching for the most frequent terms, which indicates the subtopics that receive the most attention in those fields of study (57–59). Terms such as “adolescent”, “exercise”, and “troponin-t release” were the words that were viewed in the largest and most centralized cloud, which suggests a growing interest in these terms. Authors such as Bjornan et al. (56) suggest that the largest and most central words attract more users, which may lead to new work on that topic.

For the co-authorship analysis, metadata from all of the documents were used to reveal the most productive authors, as well as the most impactful sources. The analysis showed how a large number of authors (508) contributed to the documents; being 7.43 the average number of authors per article. As Mattson et al. (60) point out, this kind of analysis is not suited to determining individual contributions, and therefore, the role of each author is not very clear. Traditionally, in multiauthor articles, the first position is occupied by the senior contributor, while the final position is reserved for the supervisor (61). The authors with the greatest impact in the studied category generally held relevant positions, either as the main author or as the supervisor. This practice is becoming increasingly common due to the influence of experimental sciences, which accord equal importance to the first and last author based on the author/director relationship (43, 51). This interpretation is known as the FLAE approach, which stands for first–last–author–emphasis (62). In this sense, our work reflects how Nie and George are referents within the context of this topic, and they represent two key figures in the research on cardiac troponin in pediatric populations.

The h-index quantifies the research performance of individual scientists, incorporating both the number and visibility of their publications (43). In the present study, an even distribution of the h-index can be seen among the most productive authors, where the number of citations that a scientific subcommunity grants to a manuscript is undoubtedly and directly related to the number of researchers that make up said subcommunity (48).

As Jung et al. (63) show, in a context where there is great interest in intensifying international collaboration within scientific practice, our work aims to present a way to measure and visualize international collaborative work at an institutional level. The remarkable collaboration among the different authors, as observed in our study, is in line with findings from studies such as those by Zhu et al. (64) and Yu et al. (49). The joint analysis of collaboration indices to determine the relationships between the different authors of the documents allows us to better interpret the structure of international scientific collaboration networks in the study category (65). The analysis has enabled us to map and identify existing collaborations within the field of cardiac troponin in pediatric populations.

Studying the publication of documents in different countries can elucidate the importance and impact of each country in an area of study. Our work shows that all of the G7 members, with the exception of Japan, are among the most productive countries in this research area. The dominance pattern of the G7 has been seen in most scientific fields, reflecting the high economic activity and academic level of these countries (66–69, 31). Most of the articles are originated in three advanced economies: China, North America, and Western Europe. Undoubtedly, these territories, with strongly developed economies and access to the most innovative and advanced research, can support research in the field of medicine and health (70, 71). In 2022, the World Health Organization (WHO) presented in its Annual Report the finding that high-income countries spent a higher percentage of their GDPs on the health sector (72). As Zheng et al. suggest (73), this pattern is similar to that of other fields of scientific research, where collaborating countries tend to be geographically correlated and revolve around the most productive countries in terms of publication output.

Regarding our citation analysis, the most productive institutions in our study are located in China, the United States, and Europe. One reason may be that larger universities provide greater opportunities for scientists to collaborate and work on similar topics, and co-authorship can lead to higher citation rates (43).

The cooperative network of research institutions reveals the distribution of research strengths in the field of cardiac troponin. The United States, England, and China have the most extensive cooperative relationships. A wide range of research has produced results similar to ours in the field of medicine and health (31, 43, 74–76, 33). In the realm of science, collaborative work and institutional and disciplinary structures face the challenge of a global context. This new research paradigm has led to initiatives such as e-Science in countries like the United Kingdom. This is a program of global collaboration in key areas of science, and focused on the development of the country's next-generation infrastructure. According to Jirotka et al. (77), initiatives such as e-Science are necessary and essential for global collaboration, enabling the broad handling of scientific data in a multidisciplinary manner.

As some authors, including Forero-Peña et al. (78) and Rojas-Montesino et al. (74), suggest, there is a strong contrast between Latin America (excluding Mexico) and Africa vs. the US, Europe, and China, since it has been found that scientific contributions from geographic areas such as Africa and Latin America represent a low percentage in the global production of research on cardiac troponins due, as these same authors suggest, to low economic investment, limited institutional development, a limited number of researchers, and the high costs of reagents and equipment—even though this percentage has increased in the last decade.

Regarding the analysis of production and institutional collaboration, as seen in the co-occurrence network, Liverpool John Moores University and Macao Polytechnic Institute take central positions with the thickest line between them, which represents a very collaborative, narrow relationship. In addition, the connecting lines between countries are intertwined, indicating that academics and institutions from each country contribute their respective strengths to eliminate academic barriers and promote academic cooperation and exchange.

Previous studies have suggested that international research collaboration is motivated by the beneficial exchange of resources, skills, and expertise, as well as by sharing the high cost of research (79). In this topic, it is worth highlighting the work published by Adams in 2013 in the journal Nature, where he claimed that “the fourth era of research” would be dominated by research driven by international collaboration. In his work, the author recommended incentivizing institutions to participate in international research to avoid the risk of marginalization and the loss of talented researchers (80).

Regarding the journals with the highest numbers of publications (Nps) on cardiac troponin in pediatric populations, we were able to observe how specific journals on pediatrics (Pediatric Cardiology, Pediatric Exercise Science, and Acta Pediatrica) were of great interest in regard to the publication of specific works on the theme of our analysis. In addition, it was possible to observe that the results obtained show that a significant number of studies were concentrated in a very specific and small core of journals. Our results are in line with other works where Bradford's Law was fulfilled (44, 43, 76, 81). One may recall that Bradford's Law is a model that estimates the exponential decrease in performance of expanding the search for references in scientific journals (82). Authors such as Highhouse et al. (83) point out that, very often, authors tend to send their papers to journals they consider to be the most important.

If we pay attention to the quartiles of the analysed publications, the vast majority were published in journals in the first and second quartiles. Publishing papers in high-impact journals (Q1 and Q2) has numerous advantages, starting with the fact that researchers who publish in high-impact journals can advance in their scientific careers and be recognized as experts in the subject or area of study (84). In addition, as indicated by Derntl (85) and Cáceres Castellano (86), publishing in high-impact journals helps researchers develop their criteria, increases self-esteem, strengthens researchers’ confidence in what they do, generates ambition to continue researching and publishing more papers, and ensures the quality of new research through peer review.

The document with the greatest number of citation, is an article published in the Journal of Applied Physiology in 2012 (87). In a study carried out by Stephan van der Zwaard et al. (88), it was concluded that the Journal of Applied Physiology had published high-impact research, being a reference within the field of exercise physiology, obtaining impact values high. It is no coincidence, then, that it has always occupied the first positions among the most relevant journals, in the two categories in which it is classified within Web of Science, which are “Physiology” and “Sports Sciences”.

Regarding future lines of research, it is proposed that researchers analyze proceedings and observe whether their inclusion produces changes in the rankings and/or in the indicators obtained in this work. It would also be interesting to analyze the performance of cardiac troponin research grants and how these translates into publications. Finally, it would be fascinating to follow the temporal evolution of the research groups already identified, as well as to detect the births of groups, the creation of new institutional collaborations, and the evolution of the impact of research in this field and its transfer to society.

### 4.1 Limitations

Even though bibliographic records have been obtained from the most relevant sources at the national and international levels, it is possible that papers have been overlooked due to failures in our search strategy. Articles and reviews were examinated, and there may be some proceedings derived from some participation or presentation at conferences that have not been included.

Second, although the choice of papers was reviewed by two investigators, biases that could stem from opinions or background knowledge were uncontrollable.

Finally, the rankings of institutions, authors, countries, and journals were based on data extracted and provided by WoSCC. It has been discussed in numerous works that there are cases in which the name of the author or the institution may be spelled differently, may be abbreviated, or the author may have affiliations with several institutions. This could lead to inaccurate productivity reports from these agencies or authors.

## 5 Conclusions

To the best of our knowledge, this is the first bibliometric analysis on the investigation of cardiac troponin and physical activity in pediatric populations. Terms such as “troponin” or “cardiac troponin” have given way to more specific terms such as “cardiac troponin-t”, “cardiac biomarkers”, and “NT-proBNP”. The number of articles has progressively increased, especially in the last decade. The vast majority of the papers were published in a specific number of journals and mainly in a certain subject area (Pediatrics). A very specific index of referring authors was observed; only three authors had 10 or more articles. The number of works carried out in collaboration with foreign institutions was high, with an important presence of institutions such as Liverpool John Moores University and Macao Polytechnic Institute. Four large groups of researchers were identified, geographically located in the USA, England, Spain, and China. A total of 508 authors, hailing from 30 countries, participated. They were affiliated with 256 institutions, and the works were published in 65 different journals from around the world.

This study contributes to a better understanding of productivity and collaboration, and it identifies the main scientific institutions, sources chosen for scientific dissemination, and the main scientists.

## Data Availability

The original contributions presented in the study are included in the article/Supplementary Material, further inquiries can be directed to the corresponding author.
